# Modulation of cortical slow oscillatory rhythm by GABA_B_ receptors: an *in vitro* experimental and computational study

**DOI:** 10.1113/JP279476

**Published:** 2020-06-18

**Authors:** Maria Perez‐Zabalza, Ramon Reig, Jesus Manrique, Daniel Jercog, Milena Winograd, Nestor Parga, Maria V. Sanchez‐Vives

**Affiliations:** ^1^ Institut d'Investigaciones Biomediques August Pi i Sunyer (IDIBAPS) Barcelona Spain; ^2^ Instituto de Neurociencias de Alicante, CSIC‐UMH San Juan de Alicante Alicante Spain; ^3^ Física Teórica Universidad Autónoma Madrid Madrid Spain; ^4^ Centro de Investigación Avanzada en Física Fundamental Universidad Autónoma de Madrid Madrid Spain; ^5^ Institució Catalana de Recerca i Estudis Avançats (ICREA) Barcelona Spain

**Keywords:** cerebral cortex, computational model, inhibition, neocortical, rhythms, slow oscillations, synchronization, Up states

## Abstract

**Key points:**

- We confirm that GABA_B_ receptors (GABA_B_‐Rs) are involved in the termination of Up‐states; their blockade consistently elongates Up‐states.
- GABA_B_‐Rs also modulate Down‐states and the oscillatory cycle, thus having an impact on slow oscillation rhythm and its regularity.
- The most frequent effect of GABA_B_‐R blockade is elongation of Down‐states and subsequent decrease of oscillatory frequency, with an increased regularity. In a quarter of cases, GABA_B_‐R blockade shortened Down‐states and increased oscillatory frequency, changes that are independent of firing rates in Up‐states.
- Our computer model provides mechanisms for the experimentally observed dynamics following blockade of GABA_B_‐Rs, for Up/Down durations, oscillatory frequency and regularity. The time course of excitation, inhibition and adaptation can explain the observed dynamics of the network.
- This study brings novel insights into the role of GABA_B_‐R‐mediated slow inhibition on the slow oscillatory activity, which is considered the default activity pattern of the cortical network.

**Abstract:**

Slow wave oscillations (SWOs) dominate cortical activity during deep sleep, anaesthesia and in some brain lesions. SWOs are composed of periods of activity (Up states) interspersed with periods of silence (Down states). The rhythmicity expressed during SWOs integrates neuronal and connectivity properties of the network and is often altered under pathological conditions. Adaptation mechanisms as well as synaptic inhibition mediated by GABA_B_ receptors (GABA_B_‐Rs) have been proposed as mechanisms governing the termination of Up states. The interplay between these two mechanisms is not well understood, and the role of GABA_B_‐Rs controlling the whole cycle of the SWO has not been described. Here we contribute to its understanding by combining *in vitro* experiments on spontaneously active cortical slices and computational techniques. GABA_B_‐R blockade modified the whole SWO cycle, not only elongating Up states, but also affecting the subsequent Down state duration. Furthermore, while adaptation tends to yield a rather regular behaviour, we demonstrate that GABA_B_‐R activation desynchronizes the SWOs. Interestingly, variability changes could be accomplished in two different ways: by either shortening or lengthening the duration of Down states. Even when the most common observation following GABA_B_‐Rs blocking is the lengthening of Down states, both changes are expressed experimentally and also in numerical simulations. Our simulations suggest that the sluggishness of GABA_B_‐Rs to follow the excitatory fluctuations of the cortical network can explain these different network dynamics modulated by GABA_B_‐Rs.

## Key points

- We confirm that GABA_B_ receptors (GABA_B_‐Rs) are involved in the termination of Up‐states; their blockade consistently elongates Up‐states.
- GABA_B_‐Rs also modulate Down‐states and the oscillatory cycle, thus having an impact on slow oscillation rhythm and its regularity.
- The most frequent effect of GABA_B_‐R blockade is elongation of Down‐states and subsequent decrease of oscillatory frequency, with an increased regularity. In a quarter of cases, GABA_B_‐R blockade shortened Down‐states and increased oscillatory frequency, changes that are independent of firing rates in Up‐states.
- Our computer model provides mechanisms for the experimentally observed dynamics following blockade of GABA_B_‐Rs, for Up/Down durations, oscillatory frequency and regularity. The time course of excitation, inhibition and adaptation can explain the observed dynamics of the network.
- This study brings novel insights into the role of GABA_B_‐R‐mediated slow inhibition on the slow oscillatory activity, which is considered the default activity pattern of the cortical network.

## Introduction

Different brain states are characterized by diverse patterns of spontaneous activity. The interplay between neuromodulators, receptors, intrinsic properties and connectivity helps to explain neuronal discharge, and as a network property, the emergence of different frequencies of oscillatory activity. During slow wave sleep, the synchronized activity of a vast number of cortical neurons contributes to the large voltage fluctuations observed during EEG recordings (Contreras & Steriade, 1995; Steriade *et al*. 2001), whereas wakefulness and rapid eye movement (REM) sleep can be identified by lower voltage amplitudes (Aserinsky & Kleitman, 1953), reflecting a decrease in the number of cortical neurons discharging simultaneously. We also know that the degree of network synchronization/desynchronization changes between different brain states and is also precisely modulated during wakefulness (Harris & Thiele, 2011; Poulet & Crochet, 2019).

The modulation of network synchronization has been related to different brain functions. During slow wave oscillations (SWOs), high levels of synchronization are involved in synaptic and cellular homeostasis, as well as memory formation (Hoffman *et al*. 2007; Diekelmann & Born, 2010; Tononi & Cirelli, 2014). It has been suggested that, during wakefulness, synchronization facilitates the transfer of information between distal neurons, providing temporal coordination for specific neuronal assemblies (Varela *et al*. 2001; Doesburg *et al*. 2010; Tononi & Cirelli, 2014). On the other hand, low levels of synchronization are observed during alert or attentional states, short‐term memory tasks, or movements, at local cortical areas (Stancák & Pfurtscheller, 1996; Klimesch *et al*. 2007; Okun & Lampl, 2008; Doesburg *et al*. 2010). Malfunctions controlling neural synchronization at different oscillatory frequencies are related to several neurological diseases such as Alzheimer's (Busche *et al*. 2015; Castano‐Prat *et al*. 2019), early ageing (Castano‐Prat *et al*. 2017), Parkinson's (Little & Brown, 2014), autism (Rubenstein & Merzenich, 2003), Williams–Beuren syndrome (Dasilva *et al*. 2020) or Down syndrome (Ruiz‐Mejias *et al*. 2016), among others. Although the control of the degree of synchronization of neural activity is essential to understand normal and pathological brain function, there remain questions regarding the basic mechanisms underlying network synchronization.

SWOs (<1 Hz) are organized into alternating periods of activity and silence: Up and Down states, respectively (Steriade *et al*. 1993). This spontaneously generated neural activity can be recorded from both isolated cortical slabs *in vivo* (Timofeev *et al*. 2000) and in cortical brain slices *in vitro* (Sanchez‐Vives & McCormick, 2000, Compte *et al*. 2008), implying that the cortical network can generate SWOs on their own (i.e. without external input). Indeed, SWOs are also expressed in clinical conditions where a ‘cortical island’ occurs as a result of a lesion (Gloor *et al*. 1977) or the perilesional area around acute ischaemic cortical stroke, where SWOs can persist for months or even years (Butz *et al*. 2004). This capability of the disconnected cortical network to generate highly similar SWOs independently from the size of the cortex involved, has led to the suggestion that this SWO is the default emergent activity pattern of the cortical network (Sanchez‐Vives & Mattia, 2014; Sanchez‐Vives *et al*. 2017). Cortical SWOs either in sleep or in deep anaesthesia are characterized by a high degree of network synchronization, where large populations of neurons are engaged, shaping the slow wave sleep cycle (Bullock & McClune, 1989; Steriade *et al*. 1993; Destexhe *et al*. 1999; Ruiz‐Mejias *et al*. 2011; Bettinardi *et al*. 2015; Tort‐Colet *et al*.^2019^). This synchronization can be explained by a combination of excitatory and inhibitory input that cortical neurons receive during Up states, which results in the depolarization of the neuron membrane potential that, in turn, often generates bursts of action potentials. On the other hand, cortical activity during Down states remains rather silent. Such patterns of active and silent cortical activity depend on the balance between recurrent excitation and local inhibition (Sanchez‐Vives & McCormick, 2000; Shu *et al*. 2003; Compte *et al*. 2009, 2003). However, the precise biophysical mechanisms underlying the inhibitory modulation of SWOs is not fully understood.

To understand spontaneous SWOs, several studies have proposed potential mechanisms responsible for their generation (transition from Down to Up states), maintenance, and termination (transition from Up to Down states). In terms of finalization, several mechanisms have been proposed that mediate the transition from Up to Down states: firing rate adaptation (Compte *et al*. 2003; Sanchez‐Vives *et al*. 2010), short‐term synaptic dynamics (Timofeev *et al*. 2000; Melamed *et al*. 2008; Benita *et al*. 2012), ATP‐dependent homeostatic mechanisms mediated by ATP‐modulated potassium (K_ATP_) channels (Cunningham *et al*. 2006), GABAergic inhibition (Chen *et al*. 2012; Lemieux *et al*. 2015; Zucca *et al*. 2017) and, more specifically, pre‐ and post‐synaptic GABA_B_ receptor (GABA_B_‐R) activation (Parga & Abbott, 2007; Mann *et al*. 2009; Wang *et al*. 2010; Craig *et al*. 2013; Sanchez‐Vives *et al*. 2020). It is plausible that more than one of these mechanisms interact and contribute to the termination of the Up‐to‐Down state transition; however, this is not yet well understood due to a paucity of experimental and modelling work addressing this issue. Although there are some indications that firing rate adaptation is at least partly responsible for the termination of Down states (i.e. generation of SWOs) (Sanchez‐Vives *et al*. 2010), it is not known to what extent this is the dominant mechanism. Interestingly, modelling work has shown that GABA_B_ dynamics have the correct timescale to contribute to the Down state transition (Parga & Abbott, 2007) and that they could interact with firing rate adaptation to modulate the termination of Up states.

Here we combined biological and computational experiments to elucidate which mechanisms underlie modulation of SWOs. More specifically, we empirically studied the role of GABA_B_‐Rs in controlling the Up‐to‐Down state transitions, Up and Down state durations and variability, and their impact on the global synchronization of spontaneous activity. We also modelled and simulated two different network behaviours and tested hypotheses in order to understand the role of each of the possible biophysical mechanisms in the modulation of SWOs; finally, we tested the predictions obtained from the experimental observations in our models.

## Methods

### Ethical approval

All experimental procedures were conducted in accordance with the European Union guidelines on the protection of vertebrates used for experimentation (Directive 2010/63/EU of the European Parliament and of the Council of 22 September 2010) and Spanish regulation RD 53/2013 on the protection of animals used for scientific purposes, approved by the ethics committee of the Hospital Clinic de Barcelona (approval number CEEA 376/11).

### Experimental procedures

To empirically study the role of GABA_B_‐Rs in the modulation of SWOs, *in vitro* experiments were performed on 37 cortical slices obtained from 13 ferrets (4–10 months old, either sex). Ferrets were deeply anaesthetized with sodium pentobarbital (40 mg/kg) and decapitated. Their brains were quickly removed and placed in ice‐cold cutting solution (4–10°C). Coronal slices (thickness: 400 µm) of the primary visual cortex (*n *= 24) and prefrontal cortex (*n *= 13) were cut on a vibratome.

A modification of the sucrose‐substitution technique developed by Aghajanian & Rasmussen (1989) was used to increase tissue viability, as in Sanchez‐Vives & McCormick (2000). After preparation, slices were placed in an interface‐style recording chamber (Fine Science Tools, Foster City, CA, USA) and bathed in ACSF containing (in mm): NaCl, 124; KCl, 2.5; MgSO_4_, 2; NaHPO_4_, 1.25; CaCl_2_, 2; NaHCO_3_, 26; and dextrose, 10; and was aerated with 95% O_2_ and 5% CO_2_ to a final pH of 7.4. Bath temperature was maintained at 34–35°C. After 2 h of recovery, the ACSF was replaced by ‘*in vivo*‐like’ ACSF (Sanchez‐Vives & McCormick, 2000) containing (in mm): KCl, 3.5; MgSO_4_, 1; CaCl_2_, 1; the remaining components were the same as those just described. Extracellular, unfiltered recordings were obtained by means of tungsten electrodes through a Neurolog system (Digitimer, Welwyn Garden City, UK) amplifier. Intra‐ and extracellular recordings were digitized, acquired using a data acquisition interface (CED) and software (Spike2) from Cambridge Electronic Design (Cambridge, UK).

To study the effects of GABA_B_ blockade on SWOs, the GABA_B_ antagonist CGP 35348 (200 µm) (Tocris) was added to the bath. A 200‐s window of recording was analysed before and after CGP 35348. The effect of GABA_B_‐R blockade was measured after an average of 17.1 ± 5.6 min after CPG‐bath application and once a steady state of the effect had been reached.

In a subset of experiments (*n *= 11), we recorded extracellularly from deep cortical layers before and after eliminating layer 1 from the cortical circuit. To isolate the circuit from long‐lasting connections, a cut was made between layers 1 and 2/3, parallel to the white matter, and two additional cuts were made perpendicular to the white matter (see Fig. 6). To study GABA_B_ blockade before and after isolating the circuit, the GABA_B_ blocker CGP 35348 (200 µm) (Tocris, Bristol, UK) was added to the bath. Baseline recordings before cutting and before CGP 35348 application were used as the control conditions.

### Spike recording and analysis

Extracellular multi‐unit recordings were obtained with 2–4 MΩ tungsten electrodes (FHC, Bowdoinham, ME, USA).

For the recorded local field potentials (LFPs), we computed the power spectral density (PSD) and estimated the multi‐unit activity (MUA) as the power change in the Fourier components at high frequencies. We assumed that the normalized LFP spectra provide a good estimate of the population firing rate, given that Fourier components at high frequencies have power densities proportional to the spiking activity of the involved neurons (Mattia & Del Giudice, 2002).

Briefly, the power spectra were computed from 5 ms sliding windows of the LFPs. MUA was estimated as the relative change of the power in the (0.2, 1.5) kHz frequency band. MUAs were logarithmically scaled in order to balance the large fluctuations of the nearby spikes. Up and Down states were singled out by setting a threshold in the log(MUA) time series. The threshold was set to 60% of the interval between the peaks in the bimodal distributions of log(MUA) corresponding to Up and Down states (Reig *et al*. 2010; Sanchez‐Vives *et al*. 2010; De Bonis *et al*. 2019). Singled‐out sets of Up and Down state durations from each recording were used to estimate the different parameters reported in the study. We calculated the following: (1) the maximum firing rate in the Up state was the peak of the average log(MUA) in the time interval (−0.5, 2.5 s) around the Up state onset; (2) the upward (Down to Up) and downward (Up to Down) transition slopes were the gradients of the linear fits of the average log(MUA) in the time intervals (−10, 25 ms) and (−25, 10 ms), respectively, around the Up state onset and offset, respectively; and (3) as a of measure SWO variability, the coefficients of variation (CV) (SD/mean) of Up state duration, Down state duration and oscillation cycle duration (Up state duration + Down state duration). All offline analyses were implemented in MATLAB (The MathWorks Inc., Natick, MA, USA).

### Statistical analysis

Before carrying out any statistical comparison, a Shapiro–Wilk test was conducted to check the normality of the data distribution. Wilcoxon's signed rank test was used to compare the effect of GABA_B_‐R blockade in the whole population, prior to the discrimination between ‘typical’/’atypical’ classification of the SWOs. Thereafter, the effect of GABA_B_‐R blockade was determined using a Kruskal–Wallis test followed by Dunn's *post hoc* test for multiple comparisons with the Bonferroni correction in the significant comparisons. All parameters studied are reported as median values together with the first (Q1) and third (Q3) quartiles. Statistical analyses were performed with IBM SPSS statistics 22 (IBM Corp., Armonk, NY, USA).

### Model and theoretical procedures

To better understand the role of GABA_B_‐Rs in the modulation of SWOs, we simulated networks of integrate‐and‐fire neurons, with the addition of a non‐linear membrane current, receiving synaptic input composed of slow and fast excitatory and inhibitory conductances (Parga & Abbott, 2007). These simulated networks consist of random connections with finite range. Each neuron is described by its membrane potential *V* which, below its threshold value, evolves according to the equation

(1) $$\begin{array}{ccc} \tau_{m}\frac{dV}{dt} & = & -g_{L}\left(V-V_{L}\right)-g_{a}\left(V-V_{a}\right)\\ & & -\;I_{syn,E}-I_{syn,I}-I_{noise}-I_{nl}. \end{array}$$

Here, *τ_m_* is the membrane time constant, *g_L_* is the leak conductance and *V_L_* is the leak reversal potential. The adaptation current, *g_a_* (*V* − *V_a_*), only affects the excitatory neurons. Its conductance, *g_a_*, decays exponentially with a time constant, *τ_a_*, until a spike is fired. When this happens, the adaptation conductance is augmented by an amount *g_a_*. *I_syn,E_* and *I_syn,I_* are the excitatory and inhibitory synaptic currents, respectively. *I_noise_* is an external noise current. *I_nl_* describes a non‐linear property of the neuron (see below). A neuron fires whenever its membrane potential *V*(*t*) reaches the spike generation threshold *V_th_*. At this point, an action potential is triggered, and the potential *V*(*t*) is reset and kept at a value *V_reset_* during a refractory period *τ_ref_*. Two excitatory (AMPA, NMDA) and two inhibitory (GABA_A_, GABA_B_) synaptic currents are included as *I_syn,E_*(*t*) = *g_AMPA_*(*V* (*t*) − *V_AMPA_*) + *g_NMDA_*(*V* (*t*) − *V_NMDA_*):

$$\begin{array}{ccc} I_{syn,I}\left(t\right) & = & g_{GABAA}\left(V\left(t\right)-V_{GABAA}\right)\\ & & +\;g_{GABA_{B}}\left(V\left(t\right)-V_{GABA_{B}}\right). \end{array}$$

When a neuron fires an action potential, the synaptic conductances of its postsynaptic neurons are modified by an amount *Δg_X_* (*X* = AMPA, NMDA, and GABA_A_, GABA_B_). Otherwise, the synaptic conductances decay exponentially, with synaptic time constant *τ_X_*. Non‐linearities characterizing NMDA and GABA_B_‐Rs are not considered; the emphasis in this model is on the time scales of the conductances. The non‐linear membrane current is a simple way of accounting for the neuron's intrinsic properties. It is described as

$$I_{nl}\left(t\right)=c\left(V\left(t\right)-V_{1}\right)\left(V\left(t\right)-V_{2}\right)\left(V\left(t\right)-V_{3}\right)$$

where *V*_1_ < *V*_2_ < *V*_3_ and *c* determines the strength of the current (for interpretations of this current see Parga & Abbott, 2007). In the absence of noise, *I_nl_* induces three fixed points, one of them being unstable. Fluctuations produced by the noise term and by the synaptic currents allow the neuron's membrane potential to alternate in a bistable fashion instead of being stuck at stable fixed‐point values. Each neuron receives independent noise currents *I_noise_* consisting of two filtered Poisson trains, one excitatory and one inhibitory. This current is parameterized by two unitary conductances (*Δg_syn,E_* and *Δg_syn,I_*), two Poisson rates and the time constants of the filter (*τ_NMDA_* and *τ_GABAB_*).

This model is not intended to be a biophysically detailed description of the cortical network; instead, it is used as a heuristic tool to explore possible explanations of the observed phenomena and to trigger experimental work.

### Parameter values

We simulated two networks (which we call the ‘typical’ and ‘atypical’ networks) to reproduce the experimentally observed data. These networks differ in their precise connectivity matrices, which were taken as independent realizations of a random architecture (as defined in the Methods) and in the values of some parameters related to synaptic and adaptation properties (see below).

We also simulated networks with parameters as those of the ‘typical’ network but with different values of the characteristic time of the adaptation current. Networks contained 4000 neurons, of which 17% were inhibitory and the rest were excitatory. Pairs of neurons separated by a distance shorter than a certain radius were connected with a probability of 2%. This radius was chosen such that, on average, each neuron was connected to 25 other neurons. The network size was 50 × 80 neurons, with periodic boundary conditions. All the neurons had a membrane time constant *τ_m_* = 20 ms and a refractory time *τ_ref_* = 5 ms. Other passive properties were distributed uniformly. The membrane threshold *V_th_* took values of −45 ± 2 mV, the reset potential *V_reset_* of −55 ± 1 mV and the leak potential *V_L_* of −68 ± 1 mV. The parameters of the non‐linear current were *c* = 0.03 mV^−2^, *V*_1_ = −72 ± 2 mV, *V*_2_ = −58 ± 2 mV and *V*_3_ = −44 ± 2 mV. AMPA and NMDA currents were present in all excitatory synapses. Similarly, we assigned GABA_A_‐Rs to 100% of the inhibitory synapses but GABA_B_‐Rs to only 70% of them.

The parameters of the noise model were: *Δg_syn,E_* = 0.09, *Δg_syn,I_* = 0.18 for the conductances and *υ_syn,E_* = 66.66 Hz, *υ_syn,I_* = 24.31 Hz for the frequency rates. We first present the values of the parameters of the synaptic and adaptation currents for the ‘atypical’ network. The synaptic time constants were *τ_AMPA_* = 2 ms, *τ_NMDA_* = 100 ms, *τ_GABAA_* = 10 ms and *τ_GABAB_* = 200 ms. All conductances are measured in units of the excitatory leak conductance (which we took as *g_E,L_* = 10 nS). *Δg_E,AMPA_* = 0.54, *Δg_E,NMDA_* = 0.04, *Δg_E,GABAA_* = 1.00 and *Δg_E,GABAB_* = 0.18. For inhibitory neurons, *Δg_I,AMPA_* = 0.57, *Δg_I,NMDA_* = 0.04, *Δg_I,GABAA_* = 0.02, *Δg_I,GABAB_* = 0.017 and *g_I,L_* = 1.4. In addition, for excitatory neurons, *Δg_a_* = 0.03, *V_a_* = −80 mV and *τ_a_* = 1900 ms. The reversal potentials for the inhibition, *V_GABAB_* and *V_GABAA_*, fall uniformly within the values −90 ± 2 and −80 ± 2 mV, respectively. *V_AMPA_* and *V_NMDA_* are both set to zero. The ‘typical’ network differs from the ‘atypical’ one only in the unitary conductances *Δg_E,NMDA_* and *Δg_I,NMDA_* (which were increased by 40%) and the adaptation characteristic time *τ_a_* (increased by 80%).

### Up and Down transition detection algorithm

This algorithm provided criteria for determining when the network moved from one state to another. The criteria can be summarized as follows. (1) Up‐to‐Down transitions: at a given time, the number of spikes of each neuron in a window of 60 ms was measured. If every cell fired less than two spikes, the transition to the Down state took place. (2) Down‐to‐Up transitions: if the percentage of neurons that fired in windows of 60 and 100 ms was at least 10% and 30%, respectively, then the transition to the Up state occurred (Luczak *et al*. 2007).

### Correlation functions and CV

We calculated spike correlograms as the average over a subpopulation of 200 randomly selected neurons of the pair‐wise correlation function

(2) $$C\left(i,j,\tau \right)=\frac{\int \rho_{i}\left(t\right)\rho_{j}\left(t-\tau \right)dt}{r_{i}r_{j}},$$

where *ρ_i_*_(_ *_j_*_)_(*t*) and *r_i_*_(_ *_j_*_)_ are the spike train and the firing rate, respectively, of neuron *i* (*j*), and τ is the time lag. Correlation functions of the currents were computed as:

(3) $$C\left({\tilde{I}}_{\alpha },{\tilde{I}}_{\beta },\tau \right)=\int {\tilde{I}}_{\alpha }\left(t\right){\tilde{I}}_{\beta }\left(t-\tau \right)dt$$

where ${\tilde{I}}_{\alpha (\beta )}$ is the population average of the current $I_{\alpha (\beta )}$. Both C(i,j,τ) and $C({\tilde{I}}_{\alpha },{\tilde{I}}_{\beta },\tau )$ were normalized to their value at their respective peaks. CVs of the duration of the Up states, the Down states and the cycle were defined as the ratio between the standard deviation and the mean (SD/mean).

### Simulation

Simulation times were typically 1200 s. Differential equations were integrated using the Euler method with an integration step of *Δt* = 0.1 ms. To obtain Fig. 8*A*, we ran eight simulations of 150 s for each value of the parameter *τ_a_*, with different realizations of the noise. Statistical errors in these graphs were computed as the SD of the values obtained in each simulation. All codes were written in C and run under the Linux operating system.

## Results

**Figure 1:**
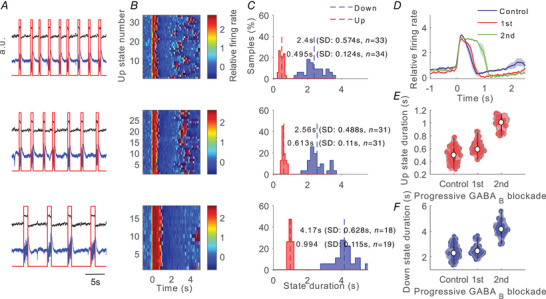
Effects of progressive inhibition blockade on slow oscillations for a single recording. Example of a ‘typical’ network *A*, raw signal (blue trace) and relative firing rate (black trace; see Methods). Up states were detected from the relative firing rate (red trace). From top to bottom: control (baseline) activity and two consecutive periods after application of 200 µm CGP 35348. Time scale is the same for all panels in *A*. For each period we analysed a single trace of 100 s. *B*, raster plots of the relative firing rate are represented for control activity and for 200 µm CGP 35348 corresponding to those in *A*. The firing rate is colour‐coded. Time scale is the same for all panels in *B*. *C*, histograms of the Up and Down duration for control activity and 200 µm CGP 35348 corresponding to those in *A*. Numerical values indicate the mean, standard deviation and number of Up and Down states detected in each period. *D*, average relative firing rate for Up states during the control and 200 µm CGP 35348 corresponding to those in *A*. The shadow corresponds to the SEM. *E*, Up state duration increases with CGP 35348. *F*, Down state duration increases with CGP 35348. Violin plots show the kernel density estimate of the data overlying the data points. The white point corresponds to the median value and vertical black lines joins the whisker ends.

The cortical network *in vitro* preserves the mechanisms to generate spontaneous rhythmic neural activity, namely SWOs, organized into Up states (active periods) and Down states (silent periods). Recordings from ferret cortical slices revealed spontaneous SWOs (Fig. 1*A*).

### Effects of GABA_B_‐R blockade on the Up/Down state cycles

The baseline frequency of the slow oscillations was 0.32 [0.25–0.39] Hz (median [first quartile–third quartile], *n* = 37) , with a duration of Up/Down states of 0.45 [0.29–0.63] s and 2.68 [2.01–3.34] s respectively.

The GABA_B_ antagonist CGP 35348 applied to the bath resulted in the gradual blockade of slow inhibition and induced several changes in the Up and Down states of the cortical slices (see Fig. 1 for a particular slice). In agreement with previous studies (Mann *et al*. 2009), a prominent and consistent change in Up and Down state properties upon GABA_B_ blockade was elongation of the Up states (Fig. 1).

**Figure 2:**
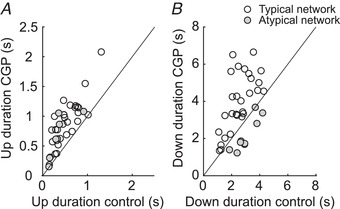
Modulation of Up and Down states by GABA_B_‐Rs *A*, scatter plot of the duration of Up states in control *versus* blockade of GABA_B_‐Rs with CGP 35348 (*n* = 37). *B*, same for Down state duration. In the two panels, the imaginary line is the one corresponding to the absence of changes (bisecting line). We define as ‘typical networks’ those where the Down state duration becomes elongated (empty circles) (*n* = 28), and ‘atypical networks’ those in which the Down states become shorter (grey‐filled circles) (*n* = 9).

**Figure 3:**
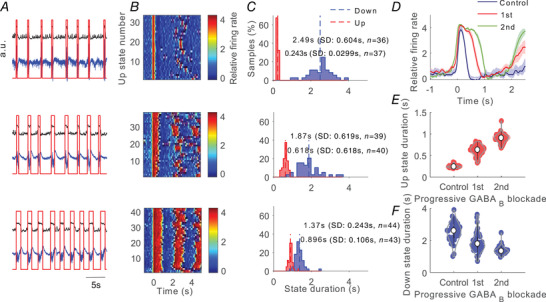
Effects of progressive inhibition blockade on slow oscillations for a single recording; example of ‘atypical’ network *A*, raw signal (blue trace), relative firing rate (black trace; see Methods) and detected Up and Down states (red trace). From top to bottom: control (baseline) activity and two consecutive periods after application of 200 µm CGP 35348. For each period we analysed a single trace of 100 s. *B*, raster plots of the relative firing rate are represented for control activity and for 200 µm CGP 35348 corresponding to those in *A*. The firing rate is colour‐coded. *C*, histograms of the Up and Down duration for control activity and 200 µm CGP 35348 corresponding to those in *A*. Numerical values indicate the mean, standard deviation and number of Up and Down states detected in each period. *D*, average relative firing rate for Up states during the control and 200 µm CGP 35348 corresponding to those in *A*. The shadow corresponds to the SEM. *E*, Up state duration increases with CGP 35348. *F*, Down state duration decreases with CGP 35348. Violin plots shows the kernel density estimate of the data overlying the data points. The white point corresponds to the median value and vertical black lines joins the whisker ends.

The Up state elongation resulting from GABA_B_ blockade was observed in 34 out of the 37 slices (Figs 1, 2 and 3*D* and *E*), while there were no changes in the remaining three cases. This elongation was on average to 182% of the original Up state duration and was observed independently of the original duration in the control (baseline) condition, which ranged between 0.14 and 1.31 s (Fig. 2*A*). The elongation of Up states following GABA_B_‐R blockade suggests that these receptors participate in the termination of the Up states, as has been previously proposed (Parga & Abbott, 2007; Mann *et al*. 2009; Wang *et al*. 2010; Craig *et al*. 2013), although it could also be secondary to the alteration of the excitatory/inhibitory balance during the Up states and subsequent modulation of the firing rate during Up states (Compte *et al*. 2003; Mattia & Sanchez‐Vives, 2012). We explore these possibilities next.

Down states were also globally elongated as a result of GABA_B_ blockade. The average elongation of the Down state (*n* = 37) was to 138% of the original value. However, when recordings were looked at individually, we observed two patterns (or groups): even though the most common effect was elongation of the Down states (∼76%, *n* = 28, Figs 1, 2*B* and Fig. 5), in some cases Down states were indeed shortened by GABA_B_ blockade with CGP 35348 (∼24%, *n* = 9, Figs 2*B*, 3 and 5). We called the first group ‘typical’ (Fig. 1) and the second one ‘atypical’ (Fig. 3), and this is how we will refer to them in the rest of the paper. To validate the distinction between the two groups of slices, we carried out discriminant analysis using as variables (1) the pre‐/post‐CGP 35348 difference in Down state duration and (2) the pre‐/post‐CGP 35348 difference in the frequency of the oscillation. Such linear discrimination analysis resulted in a reproduction of the same 9 and 28 slices corresponding to the atypical and typical types respectively.

Up and Down states are dynamically related (Compte *et al*. 2003; Sanchez‐Vives *et al*. 2010; Mattia & Sanchez‐Vives, 2012); it is therefore intriguing that the same transformation of the Up states (elongation) is followed by two different transformations of the Down states, typical elongation or atypical shortening (Fig. 2*A* and *B*). A possible functional explanation of these results could be that SWOs in ‘typical’ and ‘atypical’ networks were different to start with. To explore this possibility, we compared the properties of the SWOs displayed in control conditions, between those recordings categorized as ‘typical’ or ‘atypical’ after CGP application (see Methods). The CV of the Up state duration was significantly lower in ‘atypical’ than in ‘typical’ recordings (Table 1), demonstrating that the default SWO properties between these networks are different. Although such a difference in baseline expressed rhythm still lacks a mechanistic explanation, we further explore the possible dynamic mechanisms in our computational model below.

**Table 1:** Median (first quartile–third quartile) values of calculated parameters before GABA_B_‐R blockade for ‘typical’ group (*n* = 28) and ‘atypical’ group (*n* = 9) and *P*‐values of a Kruskal–Wallis test followed by a Dunn–Bonferroni *post hoc* test

Parameter	Median (Q1–Q3) ‘Typical’ network	Median (Q1–Q3) ‘Atypical’ network	*P*‐value
Frequency (Hz)	0.32 (0.26–0.42)	0.32 (0.23–0.35)	1
Up state duration (s)	0.48 (0.32–0.77)	0.29 (0.15–0.37)	0.367
Down state duration (s)	2.44 (1.94–3.28)	2.93(2.52–3.75)	1
CV Up–Down state cycle duration	0.36 (0.21–0.45)	0.36 (0.33–0.48)	1
CV Up state duration	0.26 (0.21–0.30)	0.17 (0.14–0.22)	0.009**
CV Down state duration	0.42 (0.26–0.51)	0.43 (0.36–0.51)	1
Maximum relative firing rate	2.43 (2.15–3.22)	2.51 (2.16–3.10)	0.9113
Down to Up transition slope (s^−1^)	18.26 (14.88–21.57)	27.27 (12.82–34.05)	0.2526
Up to Down transition slope (s^−1^)	−20.07 (−26 to −16.74)	−20.95 (−22.23 to −13.09)	1

^*^ *p* < 0.01; CV, coefficient of variation.

**Figure 4:**
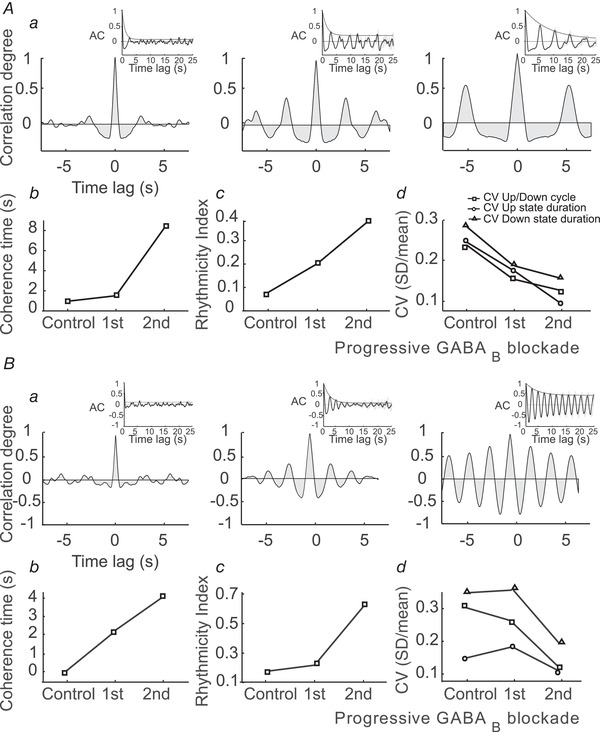
Effect of GABA_B_ blockade on the variability of slow oscillations *A*, ‘typical’ network. *a*, autocorrelograms illustrating the transformation of the emerging activity for control activity and for two consecutive periods after application of 200 µm CGP 35348 (left to right). Inset: the decay envelope of the autocorrelogram is a function of the long‐range regularity in the signal (Chatfield, 1980). *b*, measure of the decay envelope of the autocorrelogram. *c*, rhythmicity index. *d*, CV of Up/Down cycle, Up state duration and Down state duration. *B*, ‘atypical’ network. Same parameters as in *A*. The same particular cases of ‘typical’ and ‘atypical’ network are shown in Fig. 1 and Fig. 3 respectively.

Interestingly, GABA_B_ receptor blockade strikingly increased the regularity of the SWOs. This effect was obvious in the autocorrelations of the activity before and after CGP 35348, where the time constant of an exponential fitted to the peaks (coherence time; Dowse, 2009) became slower under GABA_B_‐R blockade (Fig. 4*A* and *B*, insets in *a*).

**Figure 5:**
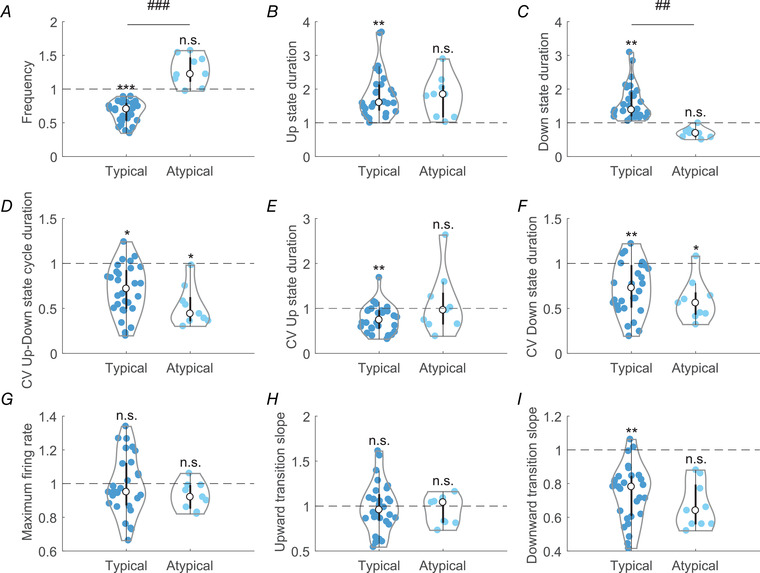
Relative changes of Up and Down state properties after blocking GABA_B_‐Rs on ‘typical’ (*n* = 28) and ‘atypical’ (*n* = 9) networks *A*, Kruskal–Wallis test followed by a Dunn–Bonferroni pairwise comparisons *post hoc* test was used to evaluate the data, *P *< 0.05 (^*^); *P *< 0.01 (^**^); *P *< 0.001 (^***^); n.s. (not significant). Comparison between groups: *P *< 0.05 (^#^); *P *< 0.01 (^##^); *P *< 0.001 (^###^) [Color figure can be viewed at wileyonlinelibrary.com]

The change in variability of the durations of Up and Down states and oscillatory cycles was quantified by the CV. The CV of both Up and Down state duration and of the oscillatory cycle decreased significantly (*P* = 0.001*, P* = 0.007 and *P* = 0.01 respectively) after GABA_B_ blockade in the ‘typical’ network (Figs 4*Ad* and 5). Compared with control values, the decrease of the CV for the ‘typical’ case was 77% and 71% for the Up and Down state durations respectively, and 70% for the complete oscillatory cycle (Fig. 5). In contrast, for the ‘atypical’ (Figs 4*Bd* and 5) network, the CV for the oscillatory cycle and Down state duration decreased (*P* = 0.018 and *P* = 0.042 respectively), but remained stable for the Up states. In the ‘atypical’ group, the decrease of the CV was 58% for the Down state duration, and 52% for the oscillatory cycle.

These results indicate that the physiological activation of GABA_B‐_Rs introduces variability and dynamic richness in the spontaneous SWOs. Hence, activation of GABA_B‐_Rs not only contributes to the elongation of the Up states, but also affects the network dynamics by controlling the duration of the Up/Down states and by disrupting the regularity of the SWOs (Fig. 5).

The firing rate during Up states is in some cases the link that explains the Up/Down state relative durations. This may be the case when the mechanisms of termination of Up states are activity‐dependent, for example the activation of sodium‐ and calcium‐dependent K^+^ currents (Compte *et al*. 2003). In cases where GABA_A_‐Rs are blocked, the decrease in fast inhibition results in high firing rates during Up states that efficiently activate sodium‐ and calcium‐dependent K^+^ currents that shorten the Up states and elongate Down states (Sanchez‐Vives *et al*. 2010). We explored if this was the case when GABA_B_‐Rs, and not GABA_A_‐Rs, were blocked. We did not find any significant difference (*P* = 0.91 for both networks) when comparing the firing rate in Up states before and after GABA_B_‐R blockade (Fig. 5*G*; *n* = 37). This suggests that the GABAergic control of firing rate in Up states occurs mostly through GABA_A_‐Rs, while the role of GABA_B_‐Rs on firing rate is negligible but noticeable on the termination of Up states and on network dynamics.

To understand the dynamics of the oscillatory activity in the cortical network it is useful to look into the transitions between states, such as the slopes of the Down‐to‐Up and Up‐to‐Down transitions. The Down‐to‐Up transition slope, which corresponds to recruitment of the local network (Reig *et al*. 2010; Sanchez‐Vives *et al*. 2010), was not affected by GABA_B_‐R blockade. Interestingly, the Up‐to‐Down (downward) transition slope decreased (Fig. 5*I*) in both ‘typical’ and ‘atypical’ networks, being significant in the ‘typical’ group (*P* = 0.004) meaning that the finalization of the SWO cycle was slower when GABA_B_‐Rs were blocked, further supporting the role of these receptors in the termination of Up states.

**Figure 6:**
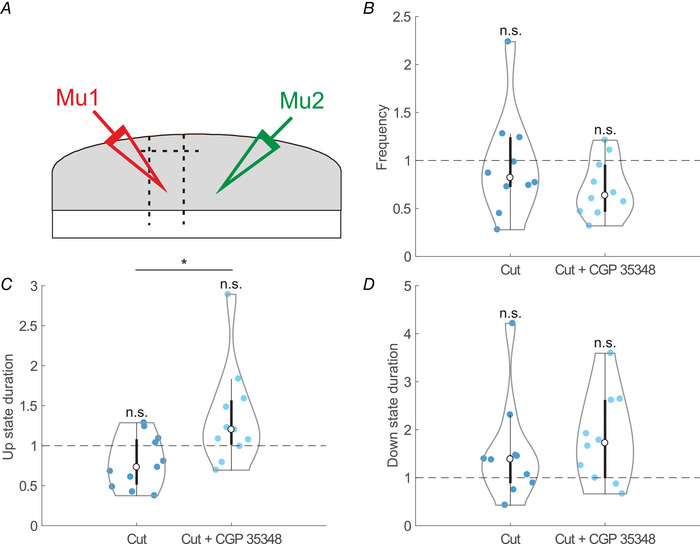
Effects of elimination of layer 1; relative changes of oscillatory frequency, Up state duration and Down state duration after cutting layer 1 and CGP application (*n* = 11) *A*, experimental sketch with double extracellular recordings. *B*, frequency after layer 1 was eliminated (Cut) and after layer 1 was eliminated and GABA_B_ blockade (Cut + CGP 35348). *C*, Up state duration. Same states as in *B*. *D*, Down state duration. Same states as in *B*. A Kruskal‐Wallis test followed by a Dunn–Bonferroni pairwise comparisons *post hoc* test was used to compare the three situations, *P *< 0.05 (^*^); *P *< 0.01 (^**^); *P *< 0.001 (^***^); n.s. (not significant). [Color figure can be viewed at wileyonlinelibrary.com]

We investigated the possible role of cortical layer 1 in modulating the spontaneous Up and Down states in infragranular layers before and after GABA_B_ blockade. To this end, we recorded spontaneous SWOs before and after eliminating layer 1 from the cortical network by cutting the slice between layers 1 and 2/3, with and without GABA_B_ blockade with CGP 35348 (Fig. 6, *n* = 11). GABA_B_ blockade resulted in a significant elongation (*P* = 0.014) of the Up states even in the absence of layer 1, similar to what occurred in control slices (Up state duration: Control 0.45 [0.27–0.55] s; layer 1 eliminated 0.31 [0.22–0.40] s; layer 1 eliminated + CGP 35348 0.50 [0.45–0.57] s). This result parallels a previous study showing that, during spontaneous oscillatory activity, GABA_B_ contributes to the Up‐to‐Down state transitions without the influence of layer 1 (Craig *et al*. 2013).

We also explored whether different circuits can display diverse effects after blocking GABA_B_‐Rs. For this, we recorded spontaneous SWOs in supra‐ and infragranular layers simultaneously with and without CGP 35348 (*n* = 16). The results did not show differences between layers after blocking GABA_B_‐Rs (Table 2). The effects on supragranular layers were not different from those described for infragranular layers in Figs 1, 2, 3, 4, 5. These results show that GABA_B_‐Rs strongly modulate the spontaneous neural activity in different layers and cortical areas as reported above.

**Table 2:** Relative changes of Up and Down state properties after blocking GABA_B_‐Rs on supragranular and infragranular layers (*n* = 16); median (first quartile–third quartile) values of normalized parameters and *P*‐values of a Kruskal–Wallis test followed by a Dunn–Bonferroni *post hoc* test

Parameter	Median (Q1–Q3) Supragranular	Median (Q1–Q3) Infragranular	*P*‐value
Frequency (Hz)	0.70 (0.50–0.79)	0.70 (0.47–0.77)	1
Up state duration (s)	1.67 (1.46–2.58)	1.58 (1.34–2.07)	1
Down state duration (s)	1.35 (1.20–2.00)	1.38 (1.17–2.07)	1
CV Up–Down state cycle duration	0.74 (0.57–0.94)	0.71 (0.51–0.93)	1
CV Up state duration	0.68 (0.61–0.99)	0.64 (0.45–0.95)	0.847
CV Down state duration	0.73 (0.55–1.00)	0.72 (0.51–0.97)	1
Maximum relative firing rate	0.98 (0.90–1.19)	0.95 (0.87–1.15)	0.9596
Down to Up transition slope (s^−1^)	0.76 (0.59–0.88)	0.98 (0.68–1.08)	0.1170
Up to Down transition slope (s^−1^)	0.82 (0.65–0.89)	0.81 (0.62–0.84)	1

In conclusion, from the experimental results we observed that blockade of GABA_B_‐Rs decreased the variability (CV) of the duration of Up and Down states as well as of the complete oscillatory cycle, suggesting that GABA_B_‐R activation plays a key role in the desynchronization of network activity. We also observed a prominent and consistent elongation of Up states as a consequence of GABA_B_‐R blockade, confirming that GABA_B_ activation participates in the termination of Up states. The fact that the slope of the Up‐to‐Down state transition became slower when GABA_B_‐Rs were blocked is in agreement with the suggested role of these receptors in Up state termination (Mann *et al*. 2009). In most cases the Up state elongation after GABA_B_ blockade occurred concurrently with an elongation of the subsequent Down states (‘typical’ network), although in a quarter of the cases the Down states shortened (‘atypical’ network). We designed a computational model of the cortical network that reproduces these observations and proposes a mechanistic explanation for them, suggesting a role for GABA_B_‐Rs in the dynamics of SWOs.

### The model: description of its basic properties

First, we present the basic features of the SWOs generated with our model. We generated two sample networks, one responding to GABA_B_‐R blockade in a ‘typical’ way and the other in an ‘atypical’ way. We next explored the most remarkable effects of GABA_B_‐Rs reported experimentally: modulation of the duration of the Up states and modulation of the regularity of the oscillations.

**Figure 7:**
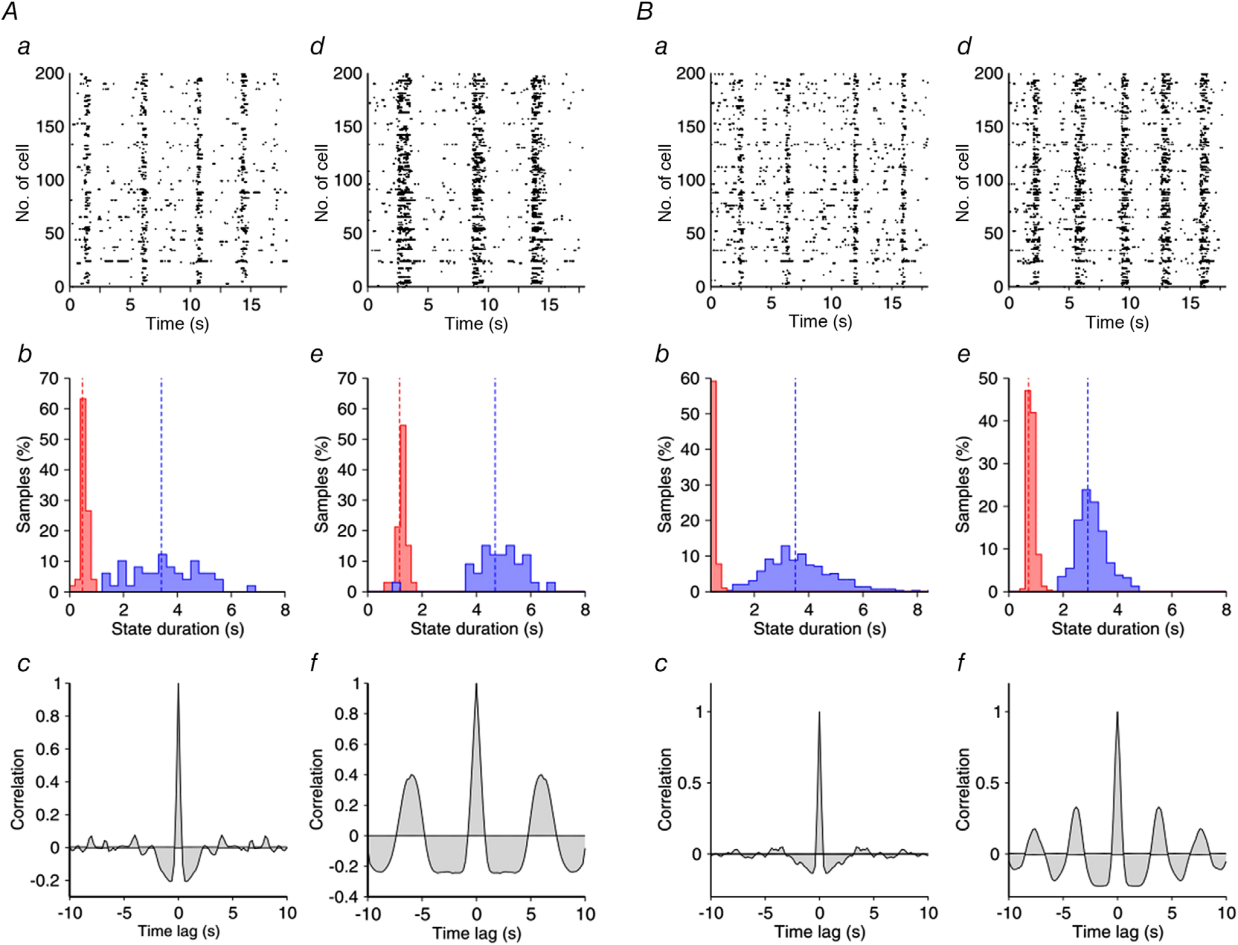
Properties of the slow oscillation in a ‘typical’ and ‘atypical’ network *A*, plots on the left refer to the control condition and on the right to the slice with blocked GABA_B_‐Rs. *a*, *d*, rastergrams. *b*, *e*, histograms of the duration of the Up (red) and Down (blue) states. Dashed lines correspond to their mean value: mean durations of Up (Down) states are 0.45 s (3.42 s) in *b* and 1.17 s (4.69 s) in *e*. *c*, *f*, spike‐train correlation functions averaged over 100 pairs of neurons. Note how the oscillation becomes more regular in the blocked condition. *B*, properties of the slow oscillation in an atypical network. Conventions are as in *A*. Mean durations of Up (Down) states are 0.36 s (3.51 s) in *b* and 0.73s (2.91s) in *e*.

We generated several sample networks with fixed connectivity but differing in the precise realization of the connectivity matrix and in the value of some parameters (see Methods for details). Two examples of networks (‘typical’ and ‘atypical’) were defined such that they had approximately the same cycle duration in the control condition (Fig. 7). For both networks, in all the generated samples and in all the slices recorded in our experiments, blocking GABA_B_‐Rs did not suppress the SWOs, and the duration of the Up states became longer. For the ‘typical’ network (Fig. 7*A*), the duration of the Down states increased as it did in the ‘typical’ experimental cases. For the ‘atypical’ network (Fig. 7*B*), the duration of the Down states decreased as it occurred in the ‘atypical’ experimental cases as well. Note that for the two networks, the histogram of the duration of Down states (panels *b* and *e* in Fig 7) had a larger dispersion when GABA_B_‐Rs were not blocked (control). To quantify the variability of the cycle and of the durations of the Up and Down states, we calculated their CV values in the network. For the two networks (Fig. 7), the CV values were [‘typical’ (‘atypical’)]: 0.29 (0.34), 0.22 (0.25) and 0.20 (0.25) for the Down state, the Up state and the whole cycle duration, respectively, in the control condition; and 0.15 (0.15), 0.10 (0.20) and 0.13 (0.15) when GABA_B_‐Rs were blocked. These values showed a substantial decrease in variability following GABA_B_ receptor blockade in both networks, especially for the duration of the Down states. Panels *c* and *f* in Fig. 7 illustrate the correlograms of the spike trains for the control and the GABA_B_‐blocked conditions.

### Explaining the modulation of variability by GABA_B_‐Rs

**Figure 8:**
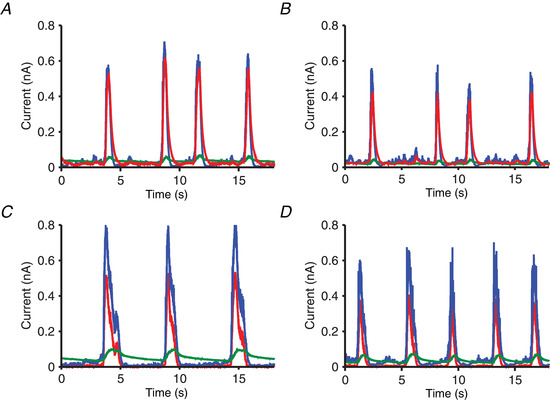
Temporal traces of the synaptic and adaptation currents Temporal traces of the population‐averaged currents for the ‘typical’ (left side) and the ‘atypical’ (right side) network. *A* and *C*, control networks; *B* and *D*, networks with the GABA_B_‐Rs blocked. Note the increasing regularity in the blocked case. Blue lines: total excitatory (AMPA plus NMDA) current. Red lines: total inhibitory (GABA_A_ plus GABA_B_) current. Green lines: adaptation current.

In our experimental results we found that the decrease in the variability of the duration of Down states and complete cycles occurred in all cases, while the decrease in Up state duration variability only occurred in typical networks. To investigate the factors responsible for the variability of the Down state duration in our model, we examined the traces of the synaptic and adaptation currents (Fig. 8*A*) for the two networks described in Fig. 7.

We first examined why SWOs were rather regular when GABA_B_‐Rs were blocked. Fluctuations of the neuronal excitation occur either from synaptic or external noise. Because in the GABA_B_‐blocked condition the inhibition is fast (GABA_A_‐mediated), fast inhibition can track these fluctuations easily (Compte *et al*. 2009; Renart *et al*. 2010) so that they do not propagate through the network unless a large population of excitatory neurons becomes active. Thus, the dynamics consist of a gradual increase of the excitation that starts during the Down state and grows until an Up state is generated (Fig. 8*B* and *D*). At this point, the fast inhibition follows this large change in the excitation but cannot suppress it. During Up states, the adaptation current progressively increases and produces the end of the Up states. Because the characteristic time of the adaptation conductance is large and spiking is rare during the Down state, this conductance decays smoothly and slowly. These mechanisms give rise to a rather regular sequence of cycles (Fig. 8*A*).

When GABA_B_‐Rs are not blocked, as in the case of the experimental control condition, these receptors produce two main effects. First, the total inhibitory current increases. A consequence of this increase is shortening of the duration of the Up states (Figs 7*Aa* and *Ba* and 8*A* and *C*). The second effect is the loss of regularity. Our experimental observations showed that when GABA_B_‐Rs were not blocked, Down states could either be longer or shorter than in the blocked condition, the second case being the most typical. In both the typical (Fig. 7*A*) and the atypical (Fig. 7*B*) simulated networks, variability was higher in the control condition, but the origin of this variability has to be explained differently because the mean duration of their Down states was related differently to the corresponding networks with blocked GABA_B_‐Rs.

For the atypical network (Fig. 7*B*), comparison of the temporal traces of the currents for the control and the blocked GABA_B_ networks obtained with simulations (Fig. 8*C* and *D*) indicate that some Up states were suppressed in the control condition. This increased the mean duration of the Down states (Fig. 7*B*) and increased their variability (from CV = 0.15 to 0.34). For the typical network (Fig. 7*A*) the temporal traces of the currents (Fig. 8*A* and *B*) indicate that, in contrast to what happened for the atypical case, now new Up states appeared with respect to the blocked condition. This also introduced a similar change in the variability of the duration of the Down states (CV increased from 0.15 to 0.29). To explain this different behaviour in typical *vs*. atypical networks, let us focus on the way that the two networks were constructed. These two networks differ in the value of only two parameters: the NMDA conductance and the characteristic time of the adaptation conductance.

**Figure 9:**
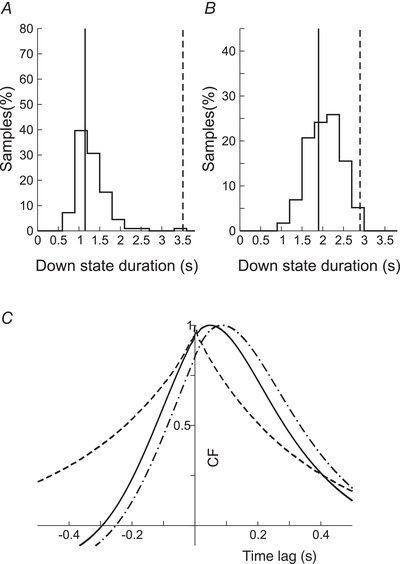
Effect of NMDA conductances on the duration of Down states *A*, histograms of the duration of the Down states in the control condition of a network with the same parameters as the ‘atypical’ one (Fig. 7*B*) but with NMDA unitary conductances increased 40%. Average Down state duration (continuous line) is 1.1 s, 68% smaller than in the original network, 3.51 s (dashed line). *B*, as in *A* but in the blocked condition. Average Down state duration (continuous line): 1.8 s, about 33% smaller than in the original network, 2.91 s (dashed line). *C*, correlation function between excitatory and inhibitory currents in the ‘atypical’ network. Full line: correlation function between total excitatory and inhibitory currents in the control condition. Dashed line: as before but in the GABA_B_‐blocked condition. Dashed‐dotted line: correlation function between total excitation and GABA_B_ component of the inhibition. CF, correlation function.

In the typical network, the NMDA unitary conductance is 40% larger than in the atypical one. To see the effect of a larger NMDA conductance on the duration of the Down states, let us consider a network identical to the atypical one (Fig. 7*B*) but with the NMDA unitary conductance increased by 40%. The stronger excitatory recurrent inputs reduced the duration of Down states in both the control (Fig. 9*A*) and the blocked GABA_B_ (Fig. 9*B*) conditions. This effect can be observed by comparing the mean duration of Down states for the modified network with the corresponding mean duration for the original atypical network (Fig. 9*A* and *B*). However, one important difference arises: in the control condition, the shortening of the duration of the Down states is about 68% while in the blocked GABA_B_ condition it is only about 33%. Note that this difference makes the original ‘atypical’ network (Fig. 7*B*) become ‘typical’, in the sense that now the duration of Down states is longer when GABA_B_‐Rs are blocked.

Why this differential shortening of Down states? The explanation can be found in the cross‐correlation function between the excitatory and inhibitory currents in the atypical network (Fig. 9*C*). In the blocked GABA_B_ condition, the tracking of excitatory currents by inhibitory ones is almost instantaneous (the peak is located at 1.0 ms). A consequence of this is that inhibitory inputs can follow excitatory ones, until they are strong enough to cause the network to arrive at an Up state. However, in the control condition, the slow dynamics of the GABA_B_‐Rs make tracking of the excitation by inhibition difficult. This is reflected in the peak of the cross‐correlation function at 46.0 ms (Fig. 9*C*, full line). This lag is 86.0 ms if only the GABA_B_ component of the inhibition is considered (dashed‐dotted line). Because the tracking mechanism is not efficient in the control condition, the shortening of Down state duration is much more pronounced than in the blocked GABA_B_ condition.

The two differences between these two sample simulated networks were NMDA conductance, which we have already described, and the time of the adaptation conductance. Both networks (Fig. 7) were selected such that they had approximately the same Down state duration in the control condition. Although the network in Fig. 9*A* and *B* is already a ‘typical’ one, the mean duration of its Down states in the control condition is shorter than that of the ‘atypical’ network in Fig. 7*B*. To obtain the ‘typical’ network in Fig. 7*A*, we then made the characteristic time of the adaptation conductance 80% larger than in the ‘atypical’ network.

In summary, a larger NMDA conductance, together with a poorer tracking in the control condition relative to the blocked condition, tends to differentially shorten the duration of the Down states, producing a network in which this duration is shorter in the control condition.

## Discussion

In this paper we have investigated the role of GABA_B_‐Rs on the slow oscillatory rhythmicity driven by alternating between Up and Down states. To that end, we combined extracellular recordings of spontaneously active cortical network slices and computational experiments to further understand the mechanisms underlying slow wave activity. We found that GABA_B_‐Rs controlled the duration of the active periods or Up states, such that their blockage elongated them, as previously described (Mann *et al*. 2009). We found that this effect was not mediated by control of the firing rate during the Up states, but by contributing to the Up to Down transition, thus controlling network synchronization. Furthermore, GABA_B_‐Rs also had an impact on the subsequent silent periods or Down states, therefore modulating the complete oscillatory cycle. Interestingly, the effect of GABA_B_ receptor blockade on the duration of the Down states can be elongation (most commonly), but also shortening. We explore in our computational model how these two opposing effects can be caused by the same intervention. The regularity of the oscillatory cycle is another parameter of the Up/Down dynamics that is modulated by GABA_B_‐Rs, such that their blockade enhances the regularity and their activation introduces dynamic richness.

Although several biophysical mechanisms explaining SWOs have been proposed, a full characterization of each one's effects and a systematic study of their interactions is lacking. The difficulty with such studies is that SWO is a spontaneous activity that emerges from the network and most mechanisms interact and globally influence the network dynamics, so the precise dissection of individual mechanisms is not an easy task. For example, a mechanism that only affects the firing rate during Up states will modify not only Up but also Down states, because they are dynamically related. Furthermore, the fact that one mechanism investigated by an external intervention (e.g. an agonist/antagonist) introduces a change in the Up/Down dynamics does not guarantee the extent of its participation under physiological conditions or its interactions with other mechanisms. It is for this reason that these mechanisms are not yet well known and also why we need the use of computational models alongside the experiments to better explore a larger parameter space and mechanistic interactions.

The blockade of GABA_B_ inhibition resulted in this study and in others (Mann *et al*. 2009; Craig *et al*. 2013) in a consistent elongation of the Up states. Were GABA_B_ blockade to result in a decreased firing rate of the network, the Up state elongation could be seen as an indirect effect. However, the absence of effect on the firing rate points to a direct role of GABA_B_ inhibition in the termination of Up states. We observed that the average elongation (both in ‘typical’ and in ‘atypical’ cases) of Up states was up to 182%, which in absolute terms is an elongation from an average of 0.51 s (control) to 0.85 s (after CGP 35348). Out of 37 cases, only three did not show an elongation of the Up state duration as a result of GABA_B_‐R blockade. All the rest elongated in a range that varied between 101% and 368%. Interestingly, this variability was independent of the original duration of Up states, which varied between 0.14 and 1.31 s, suggesting that GABA_B_ does not have a preferential role in Up state termination for either short or long Up states. Elongation was also independent of firing rate during Up states. This suggests that GABA_B_ is an independent mechanism that terminates Up states by acting in cooperation with other mechanisms, as is the case for GABA_A_‐Rs (Steriade *et al*. 1993; Chen *et al*. 2012; Lemieux *et al*. 2015; Zucca *et al*. 2017). The blockade of fast inhibition, mediated by GABA_A_‐Rs, results in increased firing rates in the Up states, which efficiently recruit activity‐dependent mechanisms, such as potassium currents, that induce the termination of Up states and shorten them (Sanchez‐Vives *et al*. 2010). The role of GABA_A_‐Rs on the termination of Up states and the initiation of the Down states suggests that they may also have a role in the so‐called Off‐periods that disrupt local causal interactions in the cortical network in unresponsive wakefulness syndrome and natural sleep (Rosanova *et al*. 2018).

The impact of GABA_B_‐R‐mediated inhibition on the variability of intervals also suggests that the participation of GABA_B_ in the termination of Up states varies depending on the functional state of the network, being lower for those states that are highly regular. In the experimental study, we found that the regularity of the cycle significantly increased when GABA_B_‐Rs were blocked. In the model, by blocking GABA_B_‐Rs the network went into an oscillatory alternation of states dominated by adaptation mechanisms, resulting in a more regular oscillatory rhythm. This shows that GABA_B_‐R activation not only plays a role in the termination of Up states, but also introduces variability in the oscillatory cycle. That the regularity of the oscillatory cycle can range from very high in deep (slow‐wave) sleep or anaesthesia to very low and chaotic during periods of transition to the awake state (Deco *et al*. 2009) suggests that different mechanisms regulate transitions between Up and Down states in different functional states (Tort‐Colet *et al*. 2019). According to our results, a reduced contribution of GABA_B_ inhibition to the dynamics of the SWOs would be expected in highly regular periods of activity such as deep sleep or anaesthesia which, according to our model, could well be regulated by adaptation mechanisms. Experiments in awake rodents have shown different degrees of network synchronization: whereas alert states are characterized by desynchronized activity, resting awake states are characterized by more synchronized activity, with slow spontaneous fluctuations (Poulet and Crochet 2019). GABA_B_‐Rs could be involved in switching between these different functional states by modulating the network synchronization. Under *in vivo* conditions, the role of GABA_B_‐Rs will also interact with other subcortical and cortical inputs and neurotransmitter effects, the influence of GABA_B_‐Rs being eventually enhanced or diminished depending on the brain state.

It has been shown that electrical stimulation of layer I is effective in terminating Up states (Mann *et al*. 2009). This effect is blocked by the GABA_B_ receptor‐blocker CGP 55845, suggesting that the Up state termination is mediated by GABA_B_ activation triggered by a subtype of interneuron in layer 1 called neurogliaform cell (Hestrin & Armstrong, 1996; Olah *et al*. 2007). However, our experimental results suggest that the termination of the spontaneous Up states is independent of layer 1‐mediated activation. Different roles for GABA_B1a_ and GABA_B1b_ subunits have also been proposed. GABA_B1a_ is preferentially located presynaptically and seems to be involved in spontaneous Up state termination; GABA_B1b_, on the other hand, is related to afferent or electrical stimulation via layer 1 activation (Craig *et al*. 2013). In our experiments, the disconnection of layer 1 from the slices did not result in the elongation of Up states; instead, Up state duration did not change or in some cases became shorter. However, applying the GABA_B_ blocker CGP 35348 after removing layer 1, Up states increased their duration as was found in slices where layer 1 was not removed (Fig. 6). This is in agreement with previous results showing that spontaneous Up‐to‐Down transitions are independent of layer 1 activation (Craig *et al*. 2013). Craig *et al*. (2013) suggested that the change in the Up‐to‐Down transition slope is mediated by presynaptic GABA_B1a_ receptor activation. In this operational framework, our model predicted that changes in NMDA conductance together with firing rate adaptation are enough to generate activity in two different networks, similar to those we observed experimentally after blocking GABA_B_‐Rs, as shown for the ‘typical’ and the ‘atypical’ cases.

We also analysed the effect of blockage of GABA_B_‐Rs in supragranular layers and the result was similar, showing that the modulation of the activity by GABA_B_ persisted in different layers and also in different cortical areas, in our case visual and prefrontal, and compatible with other authors’ and our own work (Mann *et al*. 2009; Wang *et al*. 2010; Craig *et al*. 2013).

We previously described that the partial blockade of GABA_A_‐Rs with bicuculline or gabazine (GABA_A_ receptor antagonists) increases the firing rate during Up states and decreases their regularity in active ferret cortical slices (Sanchez‐Vives *et al*. 2010). More recently, Busche *et al*. (2015) showed in wild‐type anaesthetized mice that small concentrations of gabazine (a GABA_A_ receptor antagonist) desynchronize the network activity between distal cortical areas, and treatment with benzodiazepine (a GABA_A_ agonist) restored the synchronization in a mouse model of Alzheimer's disease characterized by low levels of synchronization in the control condition. Here, we show how the blockade of GABA_B_‐Rs increases the regularity of the SWOs, suggesting that GABA_B_ can introduce desynchronization in normal conditions. On this basis, we propose a model in which GABA can modulate the network synchronization by means of the activation of GABA_A_‐Rs and GABA_B_‐Rs which generate opposing effects, synchronizing or desynchronizing the activity, respectively.

Activity‐dependent adaptation, mediated by hyperpolarizing currents, has been proposed as a critical mechanism for the termination of Up states and maintenance of Down states. Such currents would be Ca^2+^‐ and Na^+^‐dependent K^+^ currents (Compte *et al*. 2003; Sanchez‐Vives *et al*. 2010) or AMPc‐dependent potassium currents (Cunningham *et al*. 2006). Hyperpolarizing currents can also interact with other mechanisms such as synaptic depression, modulating the emerging patterns (Benita *et al*. 2012). Adaptation has also been considered in the dynamics of Up/Down states as a necessary mechanism, but in a more ample sense, such that it could include either hyperpolarizing ionic currents but also synaptic inhibition (Mattia & Sanchez‐Vives, 2012; Sanchez‐Vives, 2020). Here, in our model we considered firing rate adaptation and GABA_B_‐Rs as the two dominant biophysical factors responsible for the termination of Down states and used experimental and modelling work to investigate how they participate in slow oscillations.

The model is a generalization of the standard leaky integrate‐and‐fire model in which a non‐linear current has been included (Parga & Abbott, 2007). Adaptation is taken as a linear firing adaptation current with a characteristic time appropriate for generating oscillations with an adequate frequency. A more complete way to describe adaptation in these slices is through an activity‐dependent mechanism based on Na^+^‐ and Ca^2^‐dependent K^+^ currents (Compte *et al*. 2003). In this case, spike firing during Up states induces the accumulation of Na^+^ and Ca^2+^ ions inside the axon, which in turn causes K^+^ ions to move outside the axon, hence hyperpolarizing the neuron and terminating Up states. The duration of this hyperpolarization is determined by the time course of the decay of the Na^+^ and Ca^2+^ concentrations (Wang *et al*. 2003), giving rise to Down states. However, a modelling framework in which a simpler activity‐dependent adaptation is responsible for the Up‐to‐Down state transitions produces, in the absence of GABA_B_‐Rs, oscillations as regular as those observed in the slices. Our model considers firing rate adaptation and slow inhibition by GABA_B_ as the two biophysical elements determining the SWOs; another plausible mechanism is short‐term synaptic dynamics (Timofeev *et al*. 2000; Melamed *et al*. 2008; Benita *et al*. 2012), but we did not need it to explain the slice behaviour.

In conclusion, using *in vitro* experiments and computational models, we found that GABA_B_‐Rs critically control the synchronization of the network discharge. According to our results the decrease in GABA_B_‐R activation enhances the cycle regularity and Up state duration. This suggests that in normal conditions, GABA_B_ is a source of desynchronization in cortical activity.

## Additional information

### Competing interests

The authors declare that they have no competing interests.

### Author contributions

MVSV conceived and supervised the study. RR, MW and DJ performed the experiments. MPZ performed the formal analysis and data visualization. NP conceived and carried out the computational work with JM. All authors contributed to the paper writing. All authors have critically revised and approved the final copy of the manuscript submitted for publication. They agree to be accountable for all aspects of the work in ensuring that questions related to the accuracy or integrity of any part of the work are appropriately investigated and resolved. All persons designated as authors qualify for authorship, and all those who qualify for authorship are listed.

### Funding

This work was supported by EU H2020 Research and Innovation Programme Grant 945539 (HBP SGA3), BFU2017‐85048‐R (MINECO) and Commission for Universities and Research of the Department of Innovation, Universities, and Enterprise of the Generalitat de Catalunya ‐AGAUR‐ (IU16‐011508) to MVSV and PGC2018‐101992‐B‐100 (MINECO) to NP.

## Supporting information


**Statistical Summary Document**
