# Supplementary material for: Modulation of cortical slow oscillatory rhythm by GABAB receptors: an in vitro experimental and computational study
Source: J Physiol. 2020 Jun 18;598(16):3439–57. doi: 10.1113/JP279476 (PMC7984206; doi:10.1113/JP279476)
Supplement: Supplementary file 1 — Statistical Summary Document [file TJP-598-3439-s001.docx]

**Manuscript Title:** Modulation of cortical slow oscillatory rhythm by GABA_B_ receptors: an experimental and computational study

**Authors:** Maria Perez-Zabalza, Ramon Reig, Jesus Manrique, Milena Winograd, Nestor Parga, and Maria V. Sanchez-Vives

**Animal model used, if applicable:** Visual and prefrontal cortical slices from ferret.

**Underlying hypothesis:**

This investigation tests the effect of GABA_B_ receptors blocked in the Up and Down properties of SWO (Slow wave oscillations). We performed different comparisons: for the whole slice population (Question 1), for the two distinguish networks (Questions 2 to 5), for a subgroup of slices which layer1 was cutting (Question 6) and for a subgroup of the whole population with simultaneously double recording in infragranular and supragranular layers (Questions 7 to 10).

**Definitions of ‘*n*’:**

Question 1: *n* = 37 slices.

Question 2: *n* = 28 slices (Typical network group). Baseline Vs GABA_B_ receptors blocked.

Question 3: *n* = 9 slices (Atypical network group). Baseline Vs GABA_B_ receptors blocked.

Question 4: *n* = 28 (Typical group) and *n*= 9 (Atypical group) slices. Baseline condition. Typical Vs Atypical network.

Question 5: *n* = 28 (Typical group) and *n*= 9 (Atypical group) slices. GABA_B_ receptors blocked condition. Typical Vs Atypical network.

Question 6: *n* = 11 slices. Baseline Vs Layer 1 cutting Vs Layer 1 cutting + GABA_B_ blocked.

Question 7: *n* = 16 slices. Supragranular layers. Baseline Vs GABA_B_ blocked.

Question 8: *n* = 16 slices. Infragranular layers. Baseline Vs GABA_B_ blocked.

Question 9: *n* = 16 slices. Baseline condition. Supragranular Vs Infragranular layers.

Question 10: *n* = 16 slices. GABA_B_ receptors blocked condition. Supragranular Vs Infragranular layers.

**Statistical summary table:**

| Experimental question number* | Finding/ conclusion | Experimental location/ variable | Median value | Q1 - Q3 | n val. | P** | Units | Data comparisons | Statistical test | Any other variable | Figure in which data are presented | Comments |
| --- | --- | --- | --- | --- | --- | --- | --- | --- | --- | --- | --- | --- |
| **1 Frequency** | **Statistically significant difference** | **Baseline** | **0.32** | **0.25 – 0.39** | **37** | **0.0014** | **Hz** | **Baseline Vs CGP 35348** | **Wilcoxon signed rank** |  |  |  |
|  |  | **CGP 35348** | **0.22** | **0.17 – 0.36** |  |  |  |  |  |  |  |  |
| **1 Up state duration** | **Statistically significant difference** | **Baseline** | **0.45** | **0.29 – 0.73** |  | **1.2383E-7** | **s** |  |  |  |  |  |
|  |  | **CGP 35348** | **0.86** | **0.60 – 1.06** |  |  |  |  |  |  |  |  |
| **1 Down state duration** | **Statistically significant difference** | **Baseline** | **2.68** | **2.01 - 3.34** |  | **0.0014** | **s** |  |  |  |  |  |
|  |  | **CGP 35348** | **3.32** | **1.92 - 4.78** |  |  |  |  |  |  |  |  |
| **1 CV Up/Down cycle duration** | **Statistically significant difference** | **Baseline** | **0.36** | **0.21 – 0.46** |  | **9.8022E-7** | **au** |  |  |  |  |  |
|  |  | **CGP 35348** | **0.20** | **0.13 – 0.28** |  |  |  |  |  |  |  |  |
| **1 CV Up state duration** | **Statistically significant difference** | **Baseline** | **0.24** | **0.19 – 0.29** |  | **0.0026** | **au** |  |  |  |  |  |
|  |  | **CGP 35348** | **0.18** | **0.13 – 0.21** |  |  |  |  |  |  |  |  |
| **1 CV Down state duration** | **Statistically significant difference** | **Baseline** | **0.42** | **0.27 – 0.51** |  | **0.000003** | **au** |  |  |  |  |  |
|  |  | **CGP 35348** | **0.24** | **0.16 – 0.33** |  |  |  |  |  |  |  |  |
| 1 Maximum relative firing rate | No Statistically significant difference | Baseline | 2.48 | 2.15 – 3.18 |  | 0.1352 | au |  |  |  |  |  |
|  |  | CGP 35348 | 2.43 | 2.04 – 3.01 |  |  |  |  |  |  |  |  |
| 1 Upward transition slope | No Statistically significant difference | Baseline | 18.60 | 14.22 – 22.87 |  | 0.2610 | s^-1^ |  |  |  |  |  |
|  |  | CGP 35348 | 17.34 | 13.59 – 21.49 |  |  |  |  |  |  |  |  |
| **1 Downward transition slope** | **Statistically significant difference** | **Baseline** | **-20.20** | **-24.90 – -16.57** |  | **1.719E-7** | **s^-1^** |  |  |  |  |  |
|  |  | **CGP 35348** | **-12.85** | **-18.59 – -10.93** |  |  |  |  |  |  |  |  |
|  |  |  |  |  |  |  |  |  |  |  |  |  |
| **2 Frequency** | **Statistically significant difference** | **Baseline** | **0.32** | **0.26 – 0.42** | **28** | **0.00001** | **Hz** | **Baseline Vs CGP 35348** | **Kruskal-Wallis test followed by the Dunn-Bonferroni post hoc test.** | **Typical network** | **5A** |  |
|  |  | **CGP 35348** | **0.18** | **0.16 – 0.26** |  |  |  |  |  |  |  |  |
| **2 Up state duration** | **Statistically significant difference** | **Baseline** | **0.48** | **0.32 – 0.77** |  | **0.001** | **s** |  |  |  | **5B** |  |
|  |  | **CGP 35348** | **0.94** | **0.69 – 1.14** |  |  |  |  |  |  |  |  |
| **2 Down state duration** | **Statistically significant difference** | **Baseline** | **2.44** | **1.94 – 3.28** |  | **0.002** | **s** |  |  |  | **5C** |  |
|  |  | **CGP 35348** | **4.12** | **3.17 – 5.41** |  |  |  |  |  |  |  |  |
| **2 CV Up/Down cycle duration** | **Statistically significant difference** | **Baseline** | **0.36** | **0.21 – 0.45** |  | **0.01** | **au** |  |  |  | **5D** |  |
|  |  | **CGP 35348** | **0.19** | **0.13 – 0.297** |  |  |  |  |  |  |  |  |
| **2 CV Up state duration** | **Statistically significant difference** | **Baseline** | **0.26** | **0.21 – 0.30** |  | **0.001** | **au** |  |  |  | **5E** |  |
|  |  | **CGP 35348** | **0.18** | **0.15 – 0.21** |  |  |  |  |  |  |  |  |
| **2 CV Down state duration** | **Statistically significant difference** | **Baseline** | **0.42** | **0.26 – 0.51** |  | **0.007** | **au** |  |  |  | **5F** |  |
|  |  | **CGP 35348** | **0.24** | **0.16 – 0.35** |  |  |  |  |  |  |  |  |
| 2 Maximum relative firing rate | No Statistically significant difference | Baseline | 2.43 | 2.15 – 3.22 |  | 0.9113 | au |  |  |  | 5G |  |
|  |  | CGP 35348 | 2.44 | 2.06 – 3.05 |  |  |  |  |  |  |  |  |
| 2 Upward transition slope | No Statistically significant difference | Baseline | 18.26 | 14.88 – 21.57 |  | 0.2526 | s^-1^ |  |  |  | 5H |  |
|  |  | CGP 35348 | 16.89 | 14.09 – 19.64 |  |  |  |  |  |  |  |  |
| **2 Downward transition slope** | **Statistically significant difference** | **Baseline** | **-20.07** | **-26.00 – -16.74** |  | **0.004** | **s^-1^** |  |  |  | **5I** |  |
|  |  | **CGP 35348** | **-13.620** | **-18.68 – -11.30** |  |  |  |  |  |  |  |  |
|  |  |  |  |  |  |  |  |  |  |  |  |  |
| 3 Frequency | No Statistically significant difference | Baseline | 0.32 | 0.23 – 0.35 | 9 | 1 | Hz | Baseline Vs CGP 35348 |  | Atypical network | 5A |  |
|  |  | CGP 35348 | 0.44 | 0.27 – 0.50 |  |  |  |  |  |  |  |  |
| 3 Up state duration | No Statistically significant difference | Baseline | 0.29 | 0.15 – 0.37 |  | 0.734 | s |  |  |  | 5B |  |
|  |  | CGP 35348 | 0.61 | 0.23 – 0.95 |  |  |  |  |  |  |  |  |
| 3 Down state duration | No Statistically significant difference | Baseline | 2.93 | 2.52 – 3.75 |  | 0.601 | s |  |  |  | 5C |  |
|  |  | CGP 35348 | 1.78 | 1.52 – 3.08 |  |  |  |  |  |  |  |  |
| **3 CV Up/Down cycle duration** | **Statistically significant difference** | **Baseline** | **0.36** | **0.33 – 0.48** |  | **0.018** | **au** |  |  |  | **5D** |  |
|  |  | **CGP 35348** | **0.20** | **0.12 – 0.27** |  |  |  |  |  |  |  |  |
| 3 CV Up state duration | No Statistically significant difference | Baseline | 0.17 | 0.14 – 0.22 |  | 1 | au |  |  |  | 5E |  |
|  |  | CGP 35348 | 0.14 | 0.10 – 0.21 |  |  |  |  |  |  |  |  |
| **3 CV Down state duration** | **Statistically significant difference** | **Baseline** | **0.43** | **0.36 – 0.51** |  | **0.042** | **au** |  |  |  | **5F** |  |
|  |  | **CGP 35348** | **0.22** | **0.17 – 0.31** |  |  |  |  |  |  |  |  |
| 3 Maximum relative firing rate | No Statistically significant difference | Baseline | 2.5 | 2.16 – 3.10 |  | 0.9113 | au |  |  |  | 5G |  |
|  |  | CGP 35348 | 2.41 | 1.83 – 3.09 |  |  |  |  |  |  |  |  |
| 3 Upward transition slope | No Statistically significant difference | Baseline | 27.27 | 12.82 – 34.05 |  | 0.2526 | s^-1^ |  |  |  | 5H |  |
|  |  | CGP 35348 | 21.74 | 10.47 – 35.88 |  |  |  |  |  |  |  |  |
| 3 Downward transition slope | No Statistically significant difference | Baseline | -20.95 | -22.23 – -13.09 |  | 0.107 | s^-1^ |  |  |  | 5I |  |
|  |  | CGP 35348 | -11.15 | -18.15 – -9.26 |  |  |  |  |  |  |  |  |
|  |  |  |  |  |  |  |  |  |  |  |  |  |
| 4 Frequency | No Statistically significant difference | Typical | 0.32 | 0.26 – 0.42 | 28 | 1 | Hz | Typical Vs Atypical |  | Baseline | Table1 |  |
|  |  | Atypical | 0.32 | 0.23 – 0.35 | 9 |  |  |  |  |  |  |  |
| 4 Up state duration | No Statistically significant difference | Typical | 0.48 | 0.32 – 0.77 |  | 0.367 | s |  |  |  |  |  |
|  |  | Atypical | 0.29 | 0.15 – 0.37 |  |  |  |  |  |  |  |  |
| 4 Down state duration | No Statistically significant difference | Typical | 2.44 | 1.94 – 3.28 |  | 1 | s |  |  |  |  |  |
|  |  | Atypical | 2.93 | 2.52 – 3.75 |  |  |  |  |  |  |  |  |
| 4 CV Up/Down cycle duration | No Statistically significant difference | Typical | 0.36 | 0.21 – 0.45 |  | 1 | au |  |  |  |  |  |
|  |  | Atypical | 0.36 | 0.33 – 0.48 |  |  |  |  |  |  |  |  |
| **4 CV Up state duration** | **Statistically significant difference** | **Typical** | **0.26** | **0.21 – 0.30** |  | **0.009** | **au** |  |  |  |  |  |
|  |  | **Atypical** | **0.17** | **0.14 – 0.22** |  |  |  |  |  |  |  |  |
| 4 CV Down state duration | No Statistically significant difference | Typical | 0.42 | 0.26 – 0.51 |  | 1 | au |  |  |  |  |  |
|  |  | Atypical | 0.43 | 0.36 – 0.51 |  |  |  |  |  |  |  |  |
| 4 Maximum relative firing rate | No Statistically significant difference | Typical | 2.43 | 2.15 – 3.22 |  | 0.9113 | au |  |  |  |  |  |
|  |  | Atypical | 2.5 | 2.16 – 3.10 |  |  |  |  |  |  |  |  |
| 4 Upward transition slope | No Statistically significant difference | Typical | 18.26 | 14.88 – 21.57 |  | 0.2526 | s^-1^ |  |  |  |  |  |
|  |  | Atypical | 27.27 | 12.82 – 34.05 |  |  |  |  |  |  |  |  |
| 4 Downward transition slope | No Statistically significant difference | Typical | -20.07 | -26.00 – -16.74 |  | 1 | s^-1^ |  |  |  |  |  |
|  |  | Atypical | -20.95 | -22.23 – -13.09 |  |  |  |  |  |  |  |  |
|  |  |  |  |  |  |  |  |  |  |  |  |  |
| **5 Frequency** | **Statistically significant difference** | **Typical** | **0.18** | **0.16 – 0.26** | **28** | **0.0004** | **Hz** | **Typical Vs Atypical** |  | **CGP 35348** |  |  |
|  |  | **Atypical** | **0.44** | **0.27 – 0.50** | **9** |  |  |  |  |  |  |  |
| 5 Up state duration | No Statistically significant difference | Typical | 0.94 | 0.69 – 1.14 |  | 0.065 | s |  |  |  |  |  |
|  |  | Atypical | 0.61 | 0.23 – 0.95 |  |  |  |  |  |  |  |  |
| **5 Down state duration** | **Statistically significant difference** | **Typical** | **4.12** | **3.17 – 5.41** |  | **0.004** | **s** |  |  |  |  |  |
|  |  | **Atypical** | **0.22** | **0.17 – 0.31** |  |  |  |  |  |  |  |  |
| 5 CV Up/Down cycle duration | No Statistically significant difference | Typical | 0.19 | 0.13 – 0.297 |  | 1 | au |  |  |  |  |  |
|  |  | Atypical | 0.20 | 0.12 – 0.27 |  |  |  |  |  |  |  |  |
| 5 CV Up state duration | No Statistically significant difference | Typical | 0.18 | 0.15 – 0.21 |  | 1 | au |  |  |  |  |  |
|  |  | Atypical | 0.14 | 0.10 – 0.21 |  |  |  |  |  |  |  |  |
| 5 CV Down state duration | No Statistically significant difference | Typical | 0.24 | 0.16 – 0.35 |  | 1 | au |  |  |  |  |  |
|  |  | Atypical | 0.22 | 0.17 – 0.31 |  |  |  |  |  |  |  |  |
| 5 Maximum relative firing rate | No Statistically significant difference | Typical | 2.44 | 2.06 – 3.05 |  | 0.9113 | au |  |  |  |  |  |
|  |  | Atypical | 2.41 | 1.83 – 3.09 |  |  |  |  |  |  |  |  |
| 5 Upward transition slope | No Statistically significant difference | Typical | 16.89 | 14.09 – 19.64 |  | 0.2526 | s^-1^ |  |  |  |  |  |
|  |  | Atypical | 21.74 | 10.47 – 35.88 |  |  |  |  |  |  |  |  |
| 5 Downward transition slope | No Statistically significant difference | Typical | -13.620 | -18.68 – -11.30 |  | 1 | s^-1^ |  |  |  |  |  |
|  |  | Atypical | -11.15 | -18.15 – -9.26 |  |  |  |  |  |  |  |  |
|  |  |  |  |  |  |  |  |  |  |  |  |  |
| 6 Frequency | No Statistically significant difference | Baseline | 0.32 | 0.24 – 0.55 | 11 | 0.2507 | Hz | Control, cut and cut+cgp | Kruskal-Wallis test followed by the Dunn-Bonferroni post hoc test. |  | 6B |  |
|  |  | Cut | 0.36 | 0.14 – 0.55 |  |  |  |  |  |  |  |  |
|  |  | Cut + CGP | 0.26 | 0.17 – 0.32 |  |  |  |  |  |  |  |  |
| 6 Up state duration |  | Baseline | 0.45 | 0.27 – 0.55 | 11 | 0.281 | s | Control Vs Cut |  |  | 6C |  |
|  |  | Cut | 0.31 | 0.22 – 0.40 |  | 0.755 |  | Control Vs Cut+CGP |  |  |  |  |
|  |  | **Cut + CGP** | 0.50 | 0.45 – 0.57 |  | **0.014** |  | **Cut Vs Cut+CGP** |  |  |  |  |
| 6 Down state duration | No Statistically significant difference | Baseline | 2.61 | 1.54 – 3.45 | 11 | 0.3929 | s | Control, Cut and Cut+CGP |  |  | 6D |  |
|  |  | Cut | 2.48 | 1.36 – 6.61 |  |  |  |  |  |  |  |  |
|  |  | Cut + CGP | 3.06 | 2.52 – 5.33 |  |  |  |  |  |  |  |  |
|  |  |  |  |  |  |  |  |  |  |  |  |  |
| **7 Frequency** | **Statistically significant difference** | **Baseline** | **0.37** | **0.26 – 0.47** | **16** | **0.016** |  | **Baseline Vs CGP 35348** | **Kruskal-Wallis test followed by the Dunn-Bonferroni post hoc test.** | **Supragranular layers** |  |  |
|  |  | **CGP 35348** | **0.22** | **0.15 – 0.30** |  |  |  |  |  |  |  |  |
| **7 Up state duration** | **Statistically significant difference** | **Baseline** | **0.58** | **0.36 – 0.68** |  | 0.0001 | s |  |  |  |  |  |
|  |  | **CGP 35348** | **1.01** | **0.79 – 1.10** |  |  |  |  |  |  |  |  |
| 7 Down state duration | No Statistically significant difference | Baseline | 2.31 | 1.71 – 3.13 |  | 0.140 | s |  |  |  |  |  |
|  |  | CGP 35348 | 3.47 | 2.32 – 5.42 |  |  |  |  |  |  |  |  |
| 7 CV Up/Down cycle duration | No Statistically significant difference | Baseline | 0.20 | 0.16 – 0.39 |  | 0.334 | au |  |  |  |  |  |
|  |  | CGP 35348 | 0.16 | 0.12 – 0.21 |  |  |  |  |  |  |  |  |
| 7 CV Up state duration | No Statistically significant difference | Baseline | 0.26 | 0.21 – 0.33 |  | 0.191 | au |  |  |  |  |  |
|  |  | CGP 35348 | 0.20 | 0.13 – 0.25 |  |  |  |  |  |  |  |  |
| 7 CV Down state duration | No Statistically significant difference | Baseline | 0.24 | 0.21 – 0.45 |  | 0.268 | au |  |  |  |  |  |
|  |  | CGP 35348 | 0.19 | 0.14 – 0.27 |  |  |  |  |  |  |  |  |
| 7 Maximum relative firing rate | No Statistically significant difference | Baseline | 2.59 | 1.99 – 3.22 |  | 0.9596 | au |  |  |  |  |  |
|  |  | CGP 35348 | 2.58 | 2.18 – 3.15 |  |  |  |  |  |  |  |  |
| 7 Upward transition slope | No Statistically significant difference | Baseline | 16.92 | 11.02 – 20.71 |  | 0.1170 | s^-1^ |  |  |  |  |  |
|  |  | CGP 35348 | 11.36 | 8.35 – 18.52 |  |  |  |  |  |  |  |  |
| 7 Downward transition slope | No Statistically significant difference | Baseline | -21.90 | -24.52 – -16.56 |  | 0.06 | s^-1^ |  |  |  |  |  |
|  |  | CGP 35348 | -17.21 | -19.13 – -12.77 |  |  |  |  |  |  |  |  |
|  |  |  |  |  |  |  |  |  |  |  |  |  |
| **8 Frequency** | **Statistically significant difference** | **Baseline** | **0.36** | **0.26 –** **0.48** | **16** | **0.015** | **Hz** | **Baseline Vs CGP 35348** |  | **Infragranular layers** |  |  |
|  |  | **CGP 35348** | **0.22** | **0.15 – 0.30** |  |  |  |  |  |  |  |  |
| **8 Up state duration** | **Statistically significant difference** | **Baseline** | **0.73** | **0.49** – **0.80** |  | **0.001** | s |  |  |  |  |  |
|  |  | **CGP 35348** | **1.09** | **0.93** – **1.17** |  |  |  |  |  |  |  |  |
| 8 Down state duration | No Statistically significant difference | Baseline | 2.08 | 1.33 – 2.72 |  | 0.063 | s |  |  |  |  |  |
|  |  | CGP 35348 | 3.30 | 2.24 – 5.35 |  |  |  |  |  |  |  |  |
| 8 CV Up/Down cycle duration | No Statistically significant difference | Baseline | 0.21 | 0.17 – 0.37 |  | 0.172 | au |  |  |  |  |  |
|  |  | CGP 35348 | 0.18 | 0.13 – 0.21 |  |  |  |  |  |  |  |  |
| **8 CV Up state duration** | **Statistically significant difference** | **Baseline** | **0.27** | **0.21** – **0.34** |  | **0.002** | **au** |  |  |  |  |  |
|  |  | **CGP 35348** | **0.17** | **0.13** – **0.20** |  |  |  |  |  |  |  |  |
| 8 CV Down state duration | No Statistically significant difference | Baseline | 0.28 | 0.23 – 0.44 |  | 0.116 | au |  |  |  |  |  |
|  |  | CGP 35348 | 0.22 | 0.16 – 0 .28 |  |  |  |  |  |  |  |  |
| 8 Maximum relative firing rate | No Statistically significant difference | Baseline | 2.36 | 2.18 – 2.80 |  | 0.9596 | au |  |  |  |  |  |
|  |  | CGP 35348 | 2.44 | 2.08 – 3.08 |  |  |  |  |  |  |  |  |
| 8 Upward transition slope | No Statistically significant difference | Baseline | 16.51 | 14.88 –19.92 |  | 0.1170 | s^-1^ |  |  |  |  |  |
|  |  | CGP 35348 | 15.42 | 12.74 – 18.18 |  |  |  |  |  |  |  |  |
| 8 Downward transition slope | No Statistically significant difference | Baseline | -19.77 | -24.50 – -16.90 |  | 0.170 | s^-1^ |  |  |  |  |  |
|  |  | CGP 35348 | -14.97 | -20.16 – -11.83 |  |  |  |  |  |  |  |  |
|  |  |  |  |  |  |  |  |  |  |  |  |  |
| 9 Frequency | No Statistically significant difference | Supragranular layers | 0.37 | 0.26 – 0.47 | 16 | 1 | Hz | Supra Vs Infra |  | Baseline |  |  |
|  |  | Infragranular layers | 0.36 | 0.26 – 0.48 |  |  |  |  |  |  |  |  |
| 9 Up state duration | No Statistically significant difference | Supragranular layers | 0.58 | 0.36 – 0.68 |  | 0.73 | s |  |  |  |  |  |
|  |  | Infragranular layers | 0.73 | 0.49 – 0.80 |  |  |  |  |  |  |  |  |
| 9 Down state duration | No Statistically significant difference | Supragranular layers | 2.31 | 1.71 – 3.13 |  | 1 | s |  |  |  |  |  |
|  |  | Infragranular layers | 2.08 | 1.33 – 2.72 |  |  |  |  |  |  |  |  |
| 9 CV Up/Down cycle duration | No Statistically significant difference | Supragranular layers | 0.20 | 0.16 – 0.39 |  | 1 | au |  |  |  |  |  |
|  |  | Infragranular layers | 0.21 | 0.17 – 0.37 |  |  |  |  |  |  |  |  |
| 9 CV Up state duration | No Statistically significant difference | Supragranular layers | 0.26 | 0.21 – 0.33 |  | 1 | au |  |  |  |  |  |
|  |  | Infragranular layers | 0.27 | 0.21 – 0.34 |  |  |  |  |  |  |  |  |
| 9 CV Down state duration | No Statistically significant difference | Supragranular layers | 0.24 | 0.21 – .45 |  | 1 | au |  |  |  |  |  |
|  |  | Infragranular layers | 0.28 | 0.23 – 0.44 |  |  |  |  |  |  |  |  |
| 9 Maximum relative firing rate | No Statistically significant difference | Supragranular layers | 2.59 | 1.99 – 3.22 |  | 0.9596 | au |  |  |  |  |  |
|  |  | Infragranular layers | 2.36 | 2.18 – 2.80 |  |  |  |  |  |  |  |  |
| 9 Upward transition slope | No Statistically significant difference | Supragranular layers | 16.92 | 11.02 – 20.71 |  | 0.1170 | s^-1^ |  |  |  |  |  |
|  |  | Infragranular layers | 16.51 | 14.88 – 19.92 |  |  |  |  |  |  |  |  |
| 9 Downward transition slope | No Statistically significant difference | Supragranular layers | -21.90 | -24.52 – -16.56 |  | 1 | s^-1^ |  |  |  |  |  |
|  |  | Infragranular layers | -19.77 | -24.50 – -16.90 |  |  |  |  |  |  |  |  |
|  |  |  |  |  |  |  |  |  |  |  |  |  |
| 10 Frequency | No Statistically significant difference | Supragranular layers | 0.22 | 0.15 – 0.30 | 16 | 1 | Hz | Supra Vs Infra |  | CGP 35348 | Table2 |  |
|  |  | Infragranular layers | 0.22 | 0.15 – 0.30 |  |  |  |  |  |  |  |  |
| 10 Up state duration | No Statistically significant difference | Supragranular layers | 1.01 | 0.79 – 1.10 |  | 1 | s |  |  |  |  |  |
|  |  | Infragranular layers | 1.09 | 0.93 – 1.17 |  |  |  |  |  |  |  |  |
| 10 Down state duration | No Statistically significant difference | Supragranular layers | 3.47 | 2.32 – 5.42 |  | 1 | s |  |  |  |  |  |
|  |  | Infragranular layers | 3.30 | 2.24 – 5.35 |  |  |  |  |  |  |  |  |
| 10 CV Up/Down cycle duration | No Statistically significant difference | Supragranular layers | 0.16 | 0.12 – 0.21 |  | 1 | au |  |  |  |  |  |
|  |  | Infragranular layers | 0.18 | 0.13 – 0.21 |  |  |  |  |  |  |  |  |
| 10 CV Up state duration | No Statistically significant difference | Supragranular layers | 0.20 | 0.13 – 0.25 |  | 0.847 | au |  |  |  |  |  |
|  |  | Infragranular layers | 0.17 | 0.13 – 0.20 |  |  |  |  |  |  |  |  |
| 10 CV Down state duration | No Statistically significant difference | Supragranular layers | 0.19 | 0.14 – 0.27 |  | 1 | au |  |  |  |  |  |
|  |  | Infragranular layers | 0.22 | 0.16 – 0 .28 |  |  |  |  |  |  |  |  |
| 10 Maximum relative firing rate | No Statistically significant difference | Supragranular layers | 2.58 | 2.18 – 3.15 |  | 0.9596 | au |  |  |  |  |  |
|  |  | Infragranular layers | 2.44 | 2.08 – 3.08 |  |  |  |  |  |  |  |  |
| 10 Upward transition slope | No Statistically significant difference | Supragranular layers | 11.36 | 8.35 – 18.52 |  | 0.1170 | s^-1^ |  |  |  |  |  |
|  |  | Infragranular layers | 15.42 | 12.74 – 18.18 |  |  |  |  |  |  |  |  |
| 10 Downward transition slope | No Statistically significant difference | Supragranular layers | -17.21 | -19.13 – -12.77 |  | 1 | s^-1^ |  |  |  |  |  |
|  |  | Infragranular layers | -14.97 | -20.16 – -11.83 |  |  |  |  |  |  |  |  |

*You may use multiple lines for the same question to indicate multiple comparisons

** Authors may wish to make the text bold where p is considered significant against a stated confidence limit
